# Effect of Drying Process on the Formation of the Characteristic Flavor of Oyster (*Crassostrea hongkongensis*)

**DOI:** 10.3390/foods12112136

**Published:** 2023-05-25

**Authors:** Zhijun Wang, Hanqi Li, Wenhong Cao, Zhongqin Chen, Jialong Gao, Huina Zheng, Haisheng Lin, Xiaoming Qin

**Affiliations:** 1 College of Food Science and Technology, Guangdong Ocean University, Zhanjiang 524088, China; wangzhijun017@163.com (Z.W.); lhq350817521@163.com (H.L.); chenzhongqin@gdou.edu.cn (Z.C.); gaojl@gdou.edu.cn (J.G.); zhenghn@gdou.edu.cn (H.Z.); linhs@gdou.edu.cn (H.L.); xiaoming0502@21cn.com (X.Q.); 2 Guangdong Provincial Key Laboratory of Aquatic Products Processing and Safety, Zhanjiang 524088, China; 3 National Research and Development Branch Center for Shellfish Processing (Zhanjiang), Zhanjiang 524088, China; 4 Guangdong Provincial Engineering Technology Research Center of Seafood, Zhanjiang 524088, China; 5 Collaborative Innovation Center of Seafood Deep Processing, Dalian Polytechnic University, Dalian 116034, China

**Keywords:** *Crassostrea hongkongensis*, drying method, taste substances, volatile flavor, free amino acid

## Abstract

Oysters are nutritious and tasty but difficult to store. Drying can extend the storage period of oysters and give them a unique flavor. In this study, the effects of four drying procedures, namely, vacuum freeze drying (VFD), vacuum drying (VD), natural sun-drying (NSD), and hot air drying (HAD), on the flavor characteristics of oysters (*Crassostrea hongkongensis*) were investigated using blanched oysters as a control (CK). Results showed that HAD produced more free amino acids than the other methods, but VFD retained the most flavor nucleotides. Compared with cold drying (VFD), hot drying (VD, NSD, and HAD) increased the abundance of organic acids, betaine, and aroma substances. Glutamic acid, alanine, AMP, hexanal, octanal, heptanal, (E, E)-2,4-heptadienal, (E)-2-decenal, nonanal, etc., are defined as the characteristic flavor compounds of dried oysters, with umami, sweet, green, fatty, and fruity aromas being the main organoleptic attributes of dried oysters. Glutamic acid, glycine, betaine, IMP, pentanal, ethyl heptanoate, (E, Z)-2,4-nonadienal, 1-octen-3-one, 2-hexenal, 2-octenal, hexanal, decanal were defined as markers to distinguish different drying methods. Overall, HAD showed improved flavor qualities and characteristics and was better suited for the highly commercialized production of dried oysters.

## 1. Introduction

Oyster farming is an essential global aquaculture industry with increasing production in numerous countries worldwide [1]. According to the China Fisheries Statistical Yearbook, China’s oyster farming production in 2021 reached 581.91 × 10^4^ t [2]. Oysters are rich in protein, polysaccharides, taurine, and zinc [1,2]. Their peptides and polysaccharides also show antioxidant, anti-inflammatory, antifatigue, and antitumor functional activities, which have high nutritional and medicinal value [3,4,5]. Accordingly, oysters are favored by consumers worldwide. Oysters of superior quality that are guaranteed freshness and meet sanitary requirements are often consumed raw and are considered luxury food items owing to their delicacy [6]. However, the high moisture content of oysters and the abundance of nutrients cause easy deterioration during production, transport, and marketing due to external environments such as microorganisms and temperature, thereby losing food value [7]. Consequently, people use dry processing to lengthen the shelf life of oysters in China and other Asian countries, and the method has a long history in China [8]. Drying is an important processing method for oysters to increase their shelf life, reduce transport costs and losses due to untimely sales, and produce a dried oyster product with a unique flavor [9]. Dried oysters are primarily produced in Guangdong, China, and are an indispensable dish for Cantonese people during the holidays. In the Chinese market, consumers generally recognize dried oysters for their unique taste and flavor. Dried oysters can be eaten in many ways. They can be utilized in soup, stir-fries, and hot pots, as well as a flavor enhancer for other dishes.

The traditional method of drying oysters is primarily based on natural sun-drying (NSD), but this method has the disadvantages of low product quality and uncontrolled conditions. Hot air drying (HAD), vacuum freeze drying (VFD), and vacuum drying (VD) methods have been developed in recent years to improve drying efficiency and food quality. Owing to the different drying conditions (temperature, oxygen, and drying time), these methods have various degrees of influence on fat oxidation, protein degradation, and microbial fermentation, thereby producing different flavor substances [10,11,12]. Variations in flavor also affect product quality, and the favorable flavor is an important factor in consumer purchases. Thus, understanding the impact of drying ways on the flavor of oysters is highly valuable. Flavor comprises taste and smell. Taste contributors include free amino acids (FAAs), flavor nucleotides, and organic acids, which can give food sour, bitter, umami, and salty tastes [8,13,14]. Volatile flavors are complex and diverse in dried aquatic products. They include flavors inherent in seafood and those resulting from the oxidative degradation of proteins, lipids, and amino acids during drying [12,15]. Significant diversity exists in the flavor substances of dried aquatic products processed in different drying methods in that their flavor attributes markedly vary from those of raw seafood. Zhu et al. investigated the influence of various drying ways on *Tetraodontidae fillets* and showed that the drying method significantly affects fish flavor, with HAD producing more volatile-flavor substances and cold air drying (CAD) maintaining the stronger original flavor of the fish [16]. Zhang et al. detected the impact of diverse drying approaches on the volatile flavor in *Trachinotus blochii* fillets. They found that the fillet flavor changes depending on the drying method and that the VFD method is superior in retaining hydrocarbons and alcohols [10]. Li et al. researched the effect of different drying approaches on the flavor characteristics of *Takifugu* obscurus and discovered that HAD improves the quality of dried fish by causing higher equivalent umami concentration (EUC) values and a more pronounced pleasant odor [17]. Sun et al. clarified the diversities in the aroma features of dried *Litopenaeus vannamei* shrimps produced by diverse drying methods and showed that the best organoleptic properties of dried shrimps are obtained through microwave vacuum drying (MVD) [18].

During the drying process, the Maillard reaction, protein degradation, and lipid oxidation contribute to the unique flavor properties of the product. These reactions are interdependent and can occur simultaneously. The drying conditions play a crucial role in determining the extent of these reactions and ultimately affect the flavor quality of the dried samples. Appropriate drying conditions can improve the quality of dried oyster products, promote the structure of unique flavor substances, and offer a fundamental theory for producing high-value-added oyster products. Research on the impact of drying approaches on oyster quality is currently lacking, limiting the standardized production of high-quality dried oysters. In the present study, we investigated the characteristic flavor differences of dried oysters based on different methods (NSD, HAD, VD, and VFD). FAAs, nucleotides, organic acids, betaine, and volatile compounds were associated with the samples obtained. A combination of multiplex analysis and the OAV and TAV theories was used to determine the characteristic flavor substances of the different samples. We aimed to analyze and compare the impacts of various drying approaches on the quality of dried oysters from the perspective of flavor, to explore the differences and formation mechanisms of the key flavor of dried oysters produced by different drying methods, and to provide technical support for the development of dried oyster products with better flavor.

## 2. Materials and Methods

### 2.1. Reagents and Samples

5′-Nucleotide standards were bought from Yuanye Biotechnology Co., Ltd. (Shanghai, China). Organic acids and betaine standards were bought from Shanghai Maclean Biochemical Technology Co., Ltd. (Shanghai, China). Amino acid standards were obtained from Sigma-Aldrich Co. (St. Louis, MO, USA). High-performance liquid chromatography (HPLC)-grade acetonitrile and methanol were bought from Aladdin Reagent Co., Ltd. (Shanghai, China). 2-Methyl-3-heptanone was bought from Shanghai Macklin Biochemical Co., Ltd. (Shanghai, China). A mixed standard of n-alkanes (C5~C32) was purchased from Shanghai Anpel Experimental Technology Co., Ltd. (Shanghai, China).

**Figure 1:**
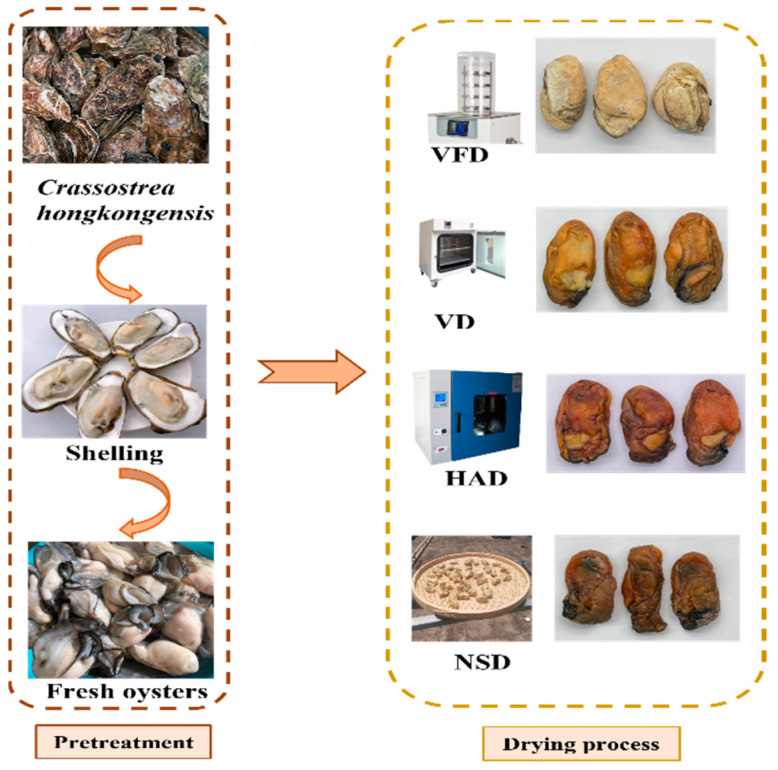
Schematic overview of the experimental program. VFD: vacuum freeze drying; VD: vacuum drying; NSD: natural sun-drying; HAD: hot air drying.

*Crassostrea hongkongensis* was purchased from the Zhanjiang Xiasan Seafood Wholesale Market in China. Fresh oysters were kept in a box with ice and transported to the laboratory within 1 h. They were shucked, selected for their uniform size and mass of 25 ± 2 g, cleaned, and boiled in a 5% salt solution (1:5, *w*/*v*) for 1 min (moisture content = 76% ± 0.5%). Blanched oysters were dried using the following four methods until the moisture content was 20% ± 2% (Figure 1). NSD was done outdoors at 33 ± 3 °C for 28 h. HAD was performed utilizing a hot-air oven at 70 °C for 11 h. VD operation was conducted in a 0.08 MPa vacuum oven at 70 °C for 12 h. VFD was performed by first freezing the oysters at −80 °C for 6 h, then freezing-drying them at 1 Pa at −50 °C for 16 h.

### 2.2. Methods

#### 2.2.1. Analysis of Nucleotides

The nucleotide content of dried oysters was identified using the method formerly described by Liu et al. [8]. In a typical procedure, 2 g of sample was homogenized with a high-speed disperser (8000 rpm) in 25 mL of 10% perchloric acid for 5 min and then centrifuged at 10,000× *g* for 20 min at 4 °C. This procedure was repeated twice, and the associated supernatants were directly neutralized with 10 mol·L^−1^ potassium hydroxide (KOH) and 1 mol·L^−1^ KOH to a pH of 6.5 and then left to stand for 30 min at 4 °C. Afterwards, the solutions were passed through a 0.45-μm filter before HPLC (Agilent 1200, Agilent Technologies, Santa Clara, CA, USA) analysis using a COSMOSIL 5C18-MS-II column under the following conditions; eluent A, methanol; eluent B, 0.05 mol·L^−1^ KH_2_PO_4_-K_2_HPO_4_; isocratic elution; flow rate, 0.7 mL/min; column temperature, 25 °C; detector wavelength, 260 nm. The identity and quantity of nucleotides were determined by comparing the retention times of each nucleotide standard and calibrating using a standard curve (R^2^ > 0.999).

#### 2.2.2. Malic Acid, Lactic Acid, Citric Acid, Succinic Acid, and Betaine Assay

Betaine was obtained via the approach of Liu et al. [6]. 1 g of the dry sample or 2 g of the fresh sample was homogenized with a high-speed disperser (8000 rpm) in 10 mL of ultrapure water for 1 min, heated at 75 °C for1 h, and centrifuged (5000× *g*, 10 min). The supernatant was then passed through a 0.45 μm filter until HPLC (Agilent 1200, Agilent Technologies, USA) analysis under the following conditions: column, Agilent Zorbax NH2; mobile phase, the eluent was 80% acetonitrile aqueous solution; isocratic elution; detection wavelength, 260 nm; and flow rate, 0.7 mL/min. Qualitative and quantitative analysis was accomplished by using retention times for betaine standards and calibration using standard curves (R^2^ > 0.999). Organic acids were identified according to the previous method by Liu et al. [6]. Additionally, 2 g of fresh or 1 g of dried sample was homogenized with a high-speed disperser (8000 rpm) in 25 mL of ultrapure water for 5 min and centrifuged (10,000× *g*, 4 °C) maintain ten minutes. The supernatant was passed through a 0.45 μm filter before HPLC (Agilent 1200, Agilent Technologies, USA) analysis under the following conditions: column, COSMOSIL 5C18-MS-II; mobile phase, 5% of 0.1% phosphoric acid solution with 95% methanol; isocratic elution; flow rate, 1 mL/min; exploration wavelength, 220 nm; column temperature, 30 °C; injection volume, 20 μL. The identity and quantity of the organic acids were determined by comparing the retention times of each organic acid standard and using the standard curve (R^2^ > 0.999) calibration.

#### 2.2.3. Analysis of FAAs and EUC

The FAAs composition of dried oysters was analyzed according to a previous method with some modifications [15]. About 0.2 g of dried oysters in 5 mL of 0.01 M HCl were ultrasonically spread for 15 min and stored at 4 °C for 2 h. The supernatant was collected from centrifugation (3000× *g*, 5 min, 4 °C) and passed through a 0.22 μm nylon membrane (Millipore, Burlington, MA, USA). After mixing 0.5 mL of the filtrate with 0.5 mL of 8% sulfosalicylic acid and storing it at 4 °C all night, the FAA profiles were analyzed with an automatic amino acid analyzer. The contents were displayed as mg/100 g oyster. The identity and quantity of amino acids were determined by comparing the retention times of each amino acid standard and using standard curve R^2^ > 0.999) calibration.

EUC was used to express the concentration of monosodium glutamate equivalent to the umami intensity given by the mixture of umami amino acids and 5′-nucleotides as follows:

(1) $$EUC=\sum a_{i}\times b_{i}+1218\left(\sum ,a_{i},\times ,b_{i}\right)\left(\sum ,a_{j},\times ,b_{j}\right)$$

where *EUC* is expressed as g MSG/100 g; *a_i_* is the concentration of the umami amino acid (Asp or Glu); *b_i_* is the related umami concentration (RUC) for every umami amino acid; *a_j_* is the concentration of umami 5′-nucleotide, *b_j_* is the RUC for every umami 5′-nucleotide (GMP, 2.5; IMP, 1; AMP, 0.18), and 1218 is the synergistic constant [8,15,19].

#### 2.2.4. Qualitative and Quantitative Analysis of Volatile Components

Variations in the dried oysters prepared through different ways of drying were analyzed using a headspace solid-phase microextraction (HS-SPME) module and gas chromatography (GC)–mass spectrometry (MS). About 5 g of fresh or 2 g of dry sample was put in a 20 mL top space vial filled with 10 μL of 50 ng/mL 2-methyl-3-heptanone as an internal standard (IS). The bottle was sealed, and the aged extractor (50/30 μm, 1 cm, DVB/CAR/PDMS fiber) was inserted into the bottle and left for 40 min at 70 °C. Then, the volatile compounds were analyzed with the GC-MS system (AOC5000-GC–MS-QP2010Plus, Shimadzu Corporation, Kyoto, Japan) coupled with a Pure-WAX quartz capillary column (30 m × 0.25 mm, 0.25 μm, Shimadzu Corporation, Japan).

The GC conditions were as follows: the actual temperature was 50 °C for 3 min, followed by ramping up to 100 °C, 140 °C, and 180 °C at 5 °C/min, 4 °C/min, and 4 °C/min, respectively, and holding time was 2, 1, and 2 min, respectively, before ramping up to 250 °C at 10 °C/min for 5 min. The carrier gas was helium at a constant flow rate of 1 mL/min, an inlet temperature of 250 °C, a pressure of 112.0 kPa, and no splitting. The MS conditions were as follows: the ion source and injector temperatures were 230 °C and 250 °C, respectively; A 70 eV electronic shock mass selection detector with an MS scan range of *m*/*z* 35–450 was used.

Qualitative analysis was carried out using the following methods. The calculated retention index (RI) was compared with the Wiley07 and NIST05 databases and with the reservation index of literature reports to identify volatile mixtures. The retention index of volatile elements was determined by comparing the reservation time of C5–C32 n-alkane standards under identical analytical conditions [20].

Quantitative analysis was performed as follows. The content of each volatile substance in each group of samples was calculated from the peak area of 2-methyl-3-heptanone at a known mass concentration using the internal-standard semiquantitative method [21,22]. The formula was as follows:

(2) $$C_{i}=\frac{10\cdot C_{is}\cdot A_{j}}{A_{is}\cdot M_{s}}$$

where *C_i_* (ng/mg) is the concentration of the compound, *C_is_* (ng/mL) is the concentration of the IS, 10 is the volume of the IS, *A_j_* is the chromatographic peak scope of the compound, *A_is_* is the IS peak scope of the mixture, and *M_s_* (mg) is the sampling mass.

#### 2.2.5. Analysis of TAV and OAV

The taste activity value (TAV) of the taste compounds in dried oysters was calculated as the ratio of concentration to the taste threshold of each compound based on Chen et al. [15].

The odor activity value (OAV) was calculated as follows [20]:

(3) $$OAV=\frac{C}{OT}$$

where *C* is the concentration of the volatile substance (ng/g), and *OT* is the odor detection threshold of the volatile compound (ng/g) measured in water.

### 2.3. Statistical Analysis

Each measurement was done thrice, and the results were expressed as mean ± standard deviation. Software SPSS 25 was used to analyze the experimental data, and one-way was used to check for significant differences (*p* < 0.05, significant difference). SIMCA 14.1 software (Umetrics, Umea, Sweden) was used for PLS-DA analysis and calculating variable importance in projection. Origin 2021 software (Origin Lab Inc., Northampton, MA, USA) was used to plot graphs.

## 3. Results and Discussion

### 3.1. Analysis of Organic Acids and Betaine

**Figure 2:**
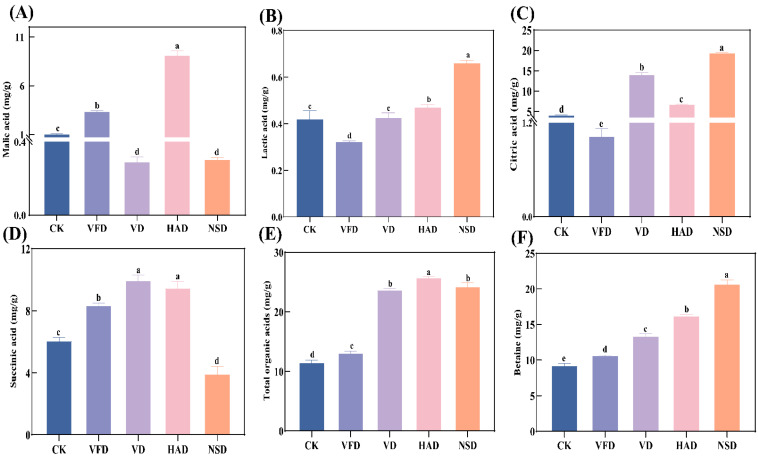
Analysis of organic acids and betaine in dried oysters under different drying conditions: (**A**) malic acid, (**B**) lactic acid, (**C**) citric acid, (**D**) succinic acid, (**E**) total organic acids, (**F**) betaine. CK: blanched oysters; VFD: vacuum freeze drying; VD: vacuum drying; NSD: natural sun-drying; HAD: hot air drying; Different letters indicate significant differences between groups (*p* < 0.05).

Figure 2 shows the analysis results of malic acid, lactic acid, citric acid, succinic acid, total organic acids, and betaine in four dried and fresh oyster species. Organic acids are essential to oyster taste, and the presence of malic, citric, lactic, and succinic acids has been reported in live oysters [6,8]. Lactic acid is an important indicator of the energy metabolism of living organisms and also contributes to taste [6,8]. Malic acid has a slightly fruity, sour taste [6]. Succinic acid contributes a strong salty and bitter taste at different concentrations in addition to its sourness [6,17]. Citric acid adds a mild, crisp acidity that contributes to the taste of oysters [6]. As shown in the graph (Figure 2), various drying ways had an effective (*p* < 0.05) impact on organic acids in oysters, with malic acid (9.06 mg/g) exhibiting a significantly higher effect on the HAD group than on the other groups (Figure 2A). The VD group had the highest concentration of succinic acid but was not statistically different from HAD (Figure 2D). Meanwhile, the NSD organization has the most citric acid (Figure 2C) content (19.29 mg/g), and lactic acid tended to increase after oyster drying (Figure 2B). Drying significantly increased the total organic acid content of oysters compared with the control group (CK), with the HAD group having the highest total organic acid content of 25.68 mg/g and the VFD group having the least change in total organic acid content. This result was similar to a previous one in which organic acids in Lentinula erodes significantly increase after HAD [23]. In the early drying stages, variations in organic acids were relative to the activity of pyruvate kinase and pyruvate dehydrogenase enzymes associated with respiratory metabolism. The increase in temperature activated the relevant enzymes in the samples and increased the organic acid content through enzymatic reactions [23]. Organic acids as precursors may be involved in enzymatic reactions, thermal degradation, Maillard reactions, and heating oxidation, which are intimately relative to the synthesis and metabolism of aromatic components, amino acids, and esters [8,24].

Betaine is a primary alkaloid compound that gives seafood a particular sweetness and contributes to its flavor [15]. In the present study, compared with fresh oysters, the betaine content of dried oysters increased significantly after drying, with the highest betaine content in the NSD group (20.60 mg/g), followed by the HAD group (16.15 mg/g), and the least change in the VFD group (Figure 2F). These outcomes were similar to those of Chen et al. Their study showed that roasting and drying processes increased the concentration of betaine in scallops. The increase in betaine may be attributed to the production of betaine from the metabolism of proteins and lipids during drying [15].

### 3.2. Analysis of FAAs

**Figure 3:**
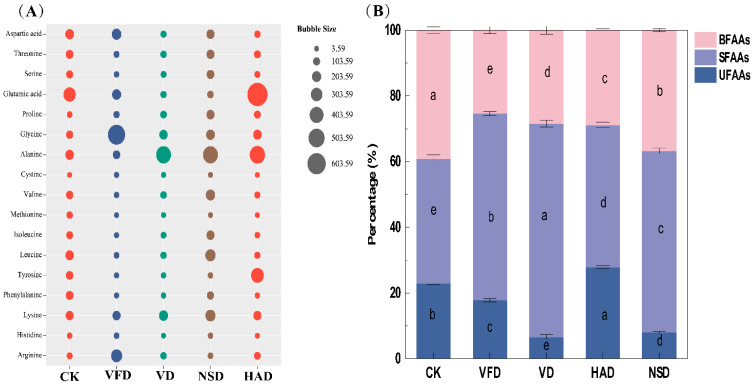
Analysis of FAAS in dried oysters under different drying conditions: (**A**) Concentration of FAAs, (**B**) Percentage of Sweet free amino acids (SFAAs), bitter free amino acids (BFAAs), and Umami amino acids (UFAAs). CK: blanched oysters; VFD: vacuum freeze drying; VD: vacuum drying; NSD: natural sun-drying; HAD: hot air drying; Different letters indicate significant differences between groups (*p* < 0.05).

Amino acid is the fundamental unit of protein and the crucial nutritional index of aquatic food [8,15]. Herein, we detected 17 FAAs in dried oysters (Figure 3A, Table S1). The total FAAs in the CK, VFD, VD, HAD, and NSD groups were 1947.46 ± 10.32, 1821.83 ± 6.21, 1400.35 ± 5.65, 2382.54 ± 1.19, and 2321.94 ± 6.02 mg/100 g, where the total FAAs in the aerobic drying group (HAD and NSD) was remarkably higher than that in the fresh oyster group, whereas the anaerobic drying group (VFD and VD) had diametrically opposite results. The drying conditions dramatically influenced the flavor quality of dried oyster products. Changes in the FAAs in oysters during desiccation and dryness were determined by combining the degradation of thermosensitive amino acids and protein metabolism [11,12]. Aerobic conditions promoted the oxidative hydrolysis of proteins, and intense proteolytic degradation increased the level of FAAs in dried oysters [12,15].

FAAs are increasingly recognized as making a significant contribution to taste and are important precursors in the formation of aroma compounds [24]. In addition to their nutritional value, amino acids affect the unique flavor profile of marine products. Alanine (Ala), aspartic acid (Asp), glycine (Gly), arginine (Arg), glutamic acid (Glu), and histidine (His) reportedly contribute significantly to the flavor of aquatic products [14,17,25]. As summarized in Table S1, the drying method significantly affected the amino acid content of dried oysters. Glutamic acid and aspartic acid were the most representative umami-tasting amino acids, and the HAD group had the highest glutamic acid content, which was 2–11.2 times higher than that of blanched oysters and other drying groups. However, high-temperature drying (VD and HAD) led to a decrease in Asp. Glutamic acid, Alanine (except VFD group), and Arg (except NSD group) were also detected at higher levels in dried oysters than in blanched oysters, a result similar to previous ones [17], indicating that dried processing can impart higher flavor quality to dried oyster products.

The impacts of the different drying methods on the constitution of umami, bitter, and sweet FAAs were further analyzed. As shown in Figure 3B, compared with fresh oysters, drying significantly enhanced the proportion of umami and sweet amino acids and decreased the relative content of bitter amino acids in oysters, which benefited the flavor of dried oysters. The HAD group had the highest relative content of umami-tasting amino acids because of the significant increase in glutamic acid.

### 3.3. Analysis of Flavor Nucleotides and Equivalent Umami Concentration (EUC)

**Figure 4:**
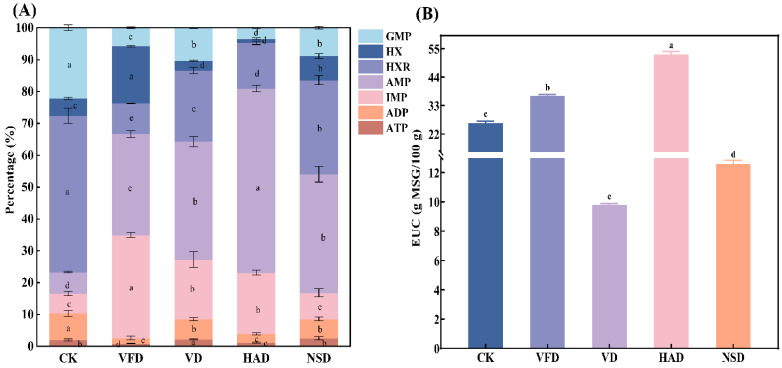
Analysis of nucleotides (**A**) and EUC (**B**) in dried oysters under different drying conditions. CK: blanched oysters; VFD: vacuum freeze drying; VD: vacuum drying; NSD: natural sun-drying; HAD: hot air drying; Different letters indicate significant differences between groups (*p* < 0.05).

Nucleotides could enhance the overall taste of the food through synergistic effects with amino acids [11]. Herein, an assessment was carried out to investigate the effect of the drying method on nucleotides and their relevant compounds, including ATP, ADP, AMP, IMP, HXR, GMP, and HX (Figure 4A, Table S1). IMP, AMP, and HXR were the major compounds in all dry groups, in compliance with the results of past research [15]. The total nucleotide content in oysters was significantly enhanced since drying treatment compared with fresh oysters, with the highest total nucleotide content in the VFD group (400.80 ± 15.79 mg/100 g), followed by the HAD group (330.71 ± 6.57 mg/100 g), and the lowest total nucleotide content in VD was only 206.86 ± 8.27 mg/100 g. Among these nucleotides, GMP, IMP, and AMP are essential sources of oyster umami and taste. The importance of GMP for the umami of seafood has been widely demonstrated [10,26,27]. However, the drying process significantly reduces the concentration of GMP in oysters, especially in the high-temperature drying group (HAD and VD). The loss of GMP may be related to its sensitivity to heat [23,28,29]. Jin et al. only detected GMP in *Coregonus peled* meat cooked at low temperatures [18]. The hot-drying process of shiitake mushrooms also led to a sudden reduction in GMP [28,29]. AMP can provide a salty and sweet taste and suppress bitterness in oysters, and IMP alone presents taste and plays a complementary role with monotonous glutamate to enhance oyster umami [8,15,29]. The trend of the sum of IMP and AMP in this study was consistent with that of total nucleotides. Subsequently, the nucleotide percentage in different groups was analyzed, and the relative content of IMP and AMP in the dried group increased significantly compared with that of the CK, with the highest relative content in the HAD group (77%), which benefited the quality of dried oysters in the HAD group.

The umami intensity in fresh and dried oysters was evaluated using the EUC theory (Figure 4B), an essential attribute of oysters. EUC refers to the synergistic umami-enhancing effect of flavor-presenting nucleotides (IMP GMP and AMP) when coexisting with glutamic acid and aspartic acid in a specific ratio to increase the umami intensity. Compared with fresh oysters, the EUC was importance significantly enhanced in the HAD and VFD groups, whereas the results were opposite in the NSD and VD groups, with the highest EUC value in the HAD (51.86 g MSG/100 g), which was 1.42–5.41 times higher compare within other groups. This finding demonstrated that HAD had a significant advantage in obtaining high EUC values, in agreement with the survey consequences of Li et al. [17].

### 3.4. Analysis of Volatile Flavor

**Figure 5:**
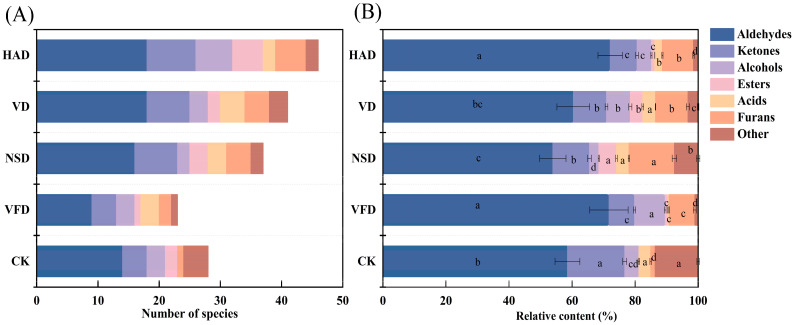
The number of species (**A**) and relative content (**B**) of various volatile fractions in dried oysters under different drying conditions. CK: blanched oysters; VFD: vacuum freeze drying; VD: vacuum drying; NSD: natural sun-drying; HAD: hot air drying; Different letters indicate significant differences between groups (*p* < 0.05).

A total of 60 volatile-flavor substances were recognized through HS-SPME/GC–MS from five groups of samples: aldehydes, alcohols, ketones, esters, hydrocarbons, furans, and acids. 28, 23, 37, 41, and 46 volatile compounds were observed in the CK, VFD, VD, NSD, and HAD groups, respectively (Table S3). The functional group classification of volatile flavor compounds was developed to analyze the variations in volatile-flavor substances in oysters after processing by different drying methods (Figure 5). Compared with the CK group, VFD resulted in the loss of some volatile flavor substances, and the hot drying method (VD, NSD, and HAD) increased the amount and concentration of volatile-flavor substances (Figure 5A). This finding indicated that dried oysters had a more intense and complex volatile flavor than fresh oysters. Similar results have been obtained in a previous study on semi-dried fish fillets by Qiu et al. [12].

Among the volatile-flavor substances, aldehydes were the main ones in raw and dried oysters, and they were higher in number and concentration than other classes of compounds. The relative content of aldehydes ranged from 54% to 72% in the five groups of samples, suggesting that aldehydes’ volatile compounds were the majority aroma components of oysters (Figure 5B). Similar results have been found in *Ostrea edulis* [30] and *Crassostrea gigas* [14,31]. Hexanal, octanal, heptanal, benzaldehyde, (E, E)-2,4-heptadienal, (E)-2-decenal, 4-pentenal, and nonanal were all discovered in dried oyster samples. Aldehydes are produced mainly by the oxidation and degradation of fatty acids [32,33]; for example, hexanal takes shape oleic, linoleic, and arachidonic acids or through degradation of other unsaturated aldehydes [34], whereas (E, E)-2,4-decadienal is an oxidation product of polyunsaturated fatty acids in the time of heat treatment. Because of the high content and low threshold of aldehydes, they have a strong fatty, orange, and grassy aroma and an effective impact on the flavor of dried-oyster food [12,35]. 

Furthermore, 4, 4, 7, 7, and 8 ketones were detected in the CK, VFD, VD, NSD, and HAD groups, respectively. Ketones are essential aroma components of dried products and are primarily produced through the thermal oxidation of polyunsaturated fatty acids or the degradation of amino acids. They can impart unique aromas and fruity flavors to the products [21,36]. Furans are produced by Maillard reactions when sugar is exposed to high temperatures and are an important source of flavor for coffee, caramel, and fruity [37,38]. Only 2-pentylfuran was detected in the CK group, whereas 2-acetylfuran, 2-ethylfuran, trans-2-(2-pentenyl) furan, and 2-5-dimethylfuran were detected in the dried oysters. The concentration of furans was much higher in dried oysters than in fresh ones.

Alcohols, which are primarily produced by fat oxidation and reduction of carbonyl compounds, have much higher flavor thresholds than aldehydes and ketones and contribute less to the overall flavor of the oyster. Nevertheless, some unsaturated alcohols have lower thresholds and contribute to the overall flavor of oysters (e.g., 1-octen-3-ol and hepten-3-ol). Notably, 1-octen-3-ol has a mushroom-like flavor and is thought to be the source of the earthy flavor in aquatic products [32,39]. 1-Octen-3-ol levels are also responsible for the fishy flavor of dried oysters and were detected in VFD, VD, and HAD dried oysters, but the highest levels were found in the VFD group.

**Figure 6:**
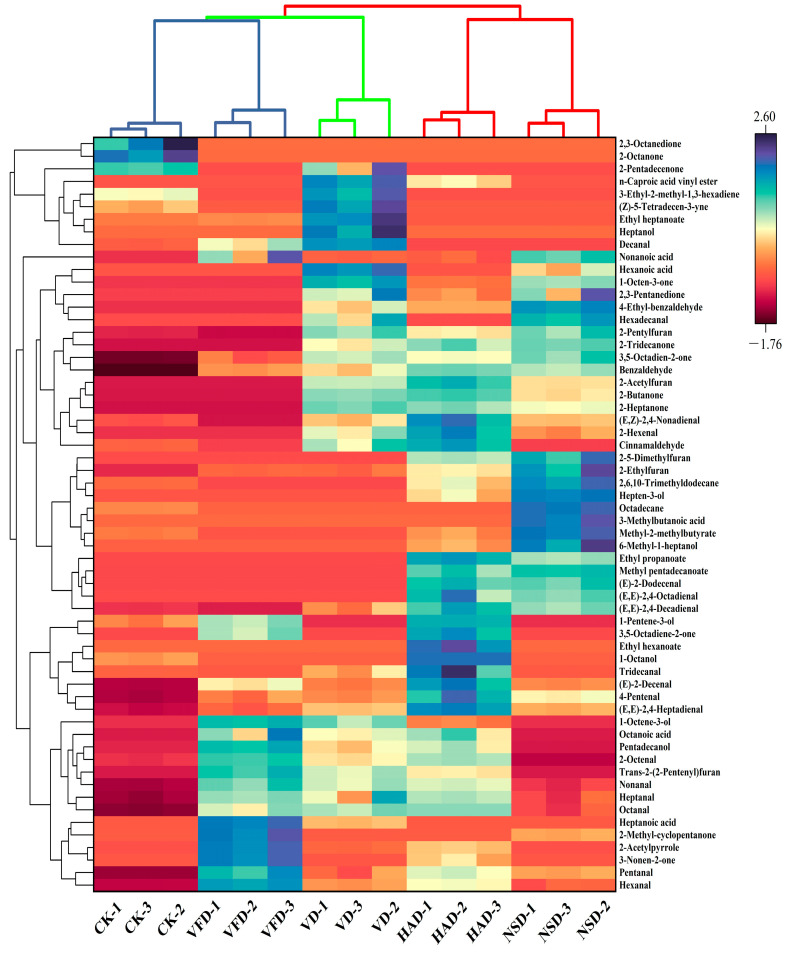
Cluster Heat Map of volatile flavor in oysters under different drying conditions. CK: blanched oysters; VFD: vacuum freeze drying; VD: vacuum drying; NSD: natural sun-drying; HAD: hot air drying.

To illustrate the impacts of diverse drying approaches on the volatile components of dry oysters, the layered cluster heat map based on the Euclidean distance way was used to study the volatile compounds of dry oysters (Figure 6). Results of the hierarchical-clustering analysis show there were remarkable differences among the samplings (*p* < 0.05). To the dendrogram, the sample columns on the horizontal axis were assigned to clusters I (CK and VFD), II (VD), and III (NSD and HAD), suggesting some similarity in the volatile flavor of the samples prepared in the NSD and HAD ways. This finding may be related to the aerobic drying environment to which they were exposed.

### 3.5. Identification of Characteristic Flavor

The amount of flavor components is insufficient to determine the characteristic flavor of a food product. The key flavor is determined by the concentration of the flavor components and the threshold value, not all of which are active characteristic flavors [21]. The OAV and TAV theories are extensively used to identify active taste and key aroma, respectively [18,40]. They have similar characteristics, and a value of TAV or OAV exceeding 1 is known to indicate that the presenting substance has a significant influence on the flavor. A greater value corresponds with a greater contribution. In the present study, 15 differential taste substances had TAV > 1 (Table S2). Each sample contained between 8 and 12 flavor compounds with TAV > 1, respectively, where Glu, Ala, succinic acid, and betaine had TAV > 1 in all samples. Gly, Arg, IMP (except the NSD group), and AMP had TAV > 1 in the dried group. Gly, Arg, and Ala provided the sweet properties of the food. Glu provided the umami. IMP and AMP contributed to the umami alone while synergistically interacting with amino acids to increase the umami intensity. These results indicated that umami and sweet taste attributes were characteristic of fresh oysters and that dried processing further enriched the intensity of this attribute.

The OAV theory was used to distinguish the volatile-flavor substances of the five groups of samples. There are 18 volatile-flavor substances had OAV values greater than 1(Table S4), including 13 aldehydes (hexanal, octanal, heptanal, (E, E)-2,4-heptadienal, (E)-2-decenal, (E, E)-2,4-decadienal, nonanal, pentanal, 2-hexenal, 2-octenal, (E, Z)-2,4-nonadienal, benzaldehyde, and decanal), 2 ketones (1-octen-3-one, 2,3-pentanedione), 1 ester (ethyl heptanoate), 1 alcohol (1-octene-3-ol), and 1 furan (2-pentylfuran). The aldehydes provide the olfactory attributes of dried oysters, such as green, fatty, and fruity aromas. 2,3-Pentanedione has a creamy smell, whereas 1-octene-3-ol and 1-octen-3-one contribute to the mushroom scent, which is also considered a fishy substance in seafood. 2-Pentylfuran has a roasted aroma, a crucial fragrance characteristic of dried oysters Aroma. The drying groups (VFD, VD, NSD, HAD) contained between 10 and 17 substances with an OAV greater than 1. The HAD group had the highest number of substances with an OAV greater than 1, with the addition of heptanal, pentanal, 2-hexenal, (E, Z)-2,4-nonadienal, benzaldehyde, 1-octen-3-one, 2,3-pentanedione, 1-octene-3-ol, and ethyl hexanoate compared to CK. This was followed by the VD group, which contained 16 species, and the VFD group, which had the fewest substances with OVA greater than 1, with only 10 species. This result indicates that high-temperature drying increased the richness of the dried oyster flavor characteristics.

**Figure 7:**
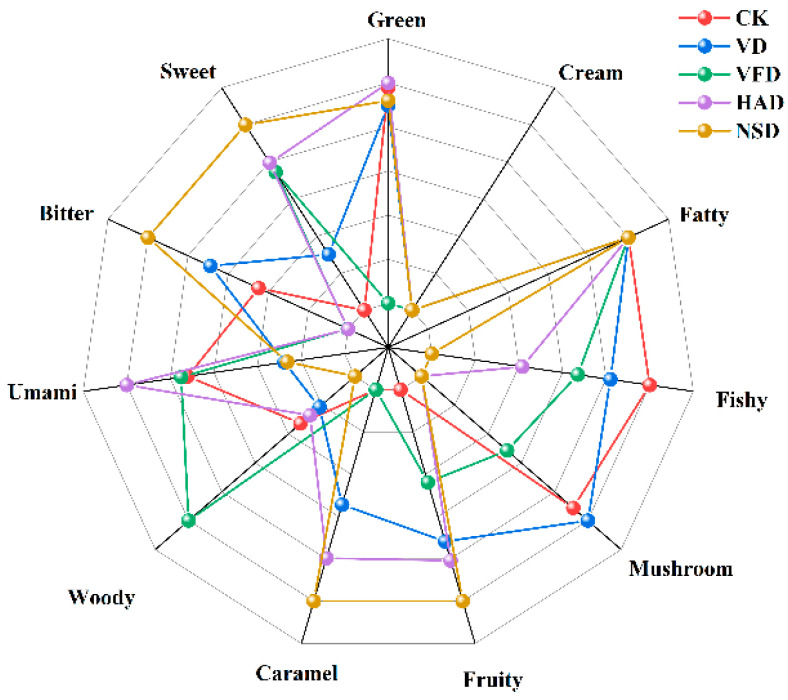
Radar plot of the characteristic flavor in dried oysters under different drying conditions. CK: blanched oysters; VFD: vacuum freeze drying; VD: vacuum drying; NSD: sun drying; HAD: hot air drying.

To visually distinguish the characteristic flavor of the five samples, we developed characteristic flavor models for the 11 sensory attributes of umami, bitter, sweet, fatty, caramel, fruity, green, orange, fishy, woody, and mushroom based on substances with TAV > 1 and OAV > 1 (The OAV of substances with the same flavor are added up and finally normalized). As shown in Figure 7, the different drying approaches have a practical influence on the characteristic flavor of the dried oysters. NSD has the highest sweetness, bitterness, fruit, and caramel properties. HAD has the highest umami and lowest bitterness. The woody of VFD is significantly higher than other drying methods. The fishy and mushroom flavor of the VD is most noticeable in the dry-aged group. Overall, these dried oysters prepared by HAD had better flavor attributes.

### 3.6. Diversified Analysis

**Figure 8:**
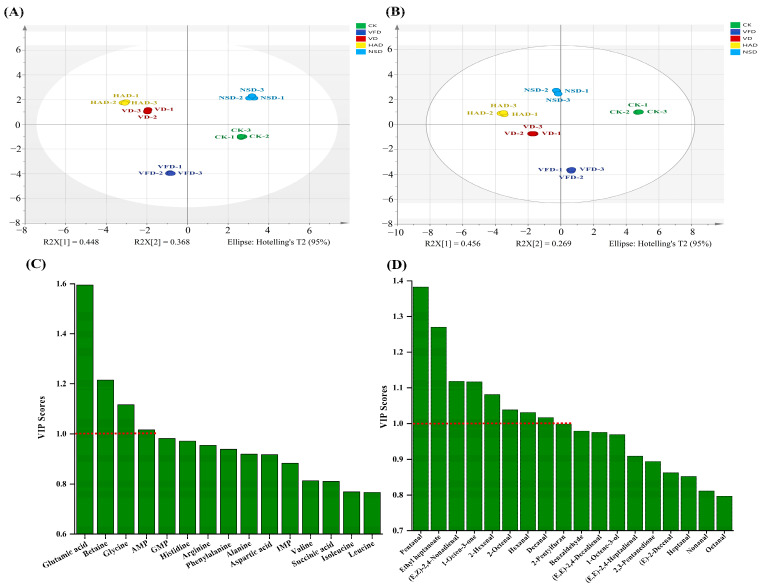
PLS-DA score plots (**A**) and (**C**) VIP scores obtained by modeling taste substances in dried oysters under different drying conditions; PLS-DA score plots (**B**) and (**D**) VIP scores obtained by modeling volatile substances in dried oysters under different drying conditions. CK: blanched oysters; VFD: vacuum freeze drying; VD: vacuum drying; NSD: natural sun-drying; HAD: hot air drying.

To focus on the differences in the characteristic flavor of various dried oysters, separate PLS-DA models were developed based on active taste substances and active odor substances (Figure 8). PLS-DA, a statistical method of supervised discriminant analysis, which is different from PCA, could actually interpret the observed values and accomplish the prediction of the corresponding variables [18,20]. The characteristics of the taste and aroma of the five samples can be distinguished separately and effectively, with the accumulative contribution of the first two main elements components reaching 81.6% and 72.5%, respectively. In PLS-DA models, the reliability and predictive power of the model can be evaluated by R^2^ and Q^2^, with R^2^ and Q^2^ above 0.5 indicating an acceptable model fit and values closer to 1 indicating a stronger predictive power. Both PLS-DA models in this study met the requirement (R^2^(X) = 0.994, R^2^(Y) = 0.996, Q^2^ = 0.993; R^2^(X) = 0.991, R^2^(Y) = 0.995, Q^2^ = 0.988). Moreover, since 200 permutation tests, the intersection of the Q^2^ regression line with the vertical axis was less than zero, showing the model was not overfitted and the model validation was effective. Therefore, these results could be used to analyze the signature substances of different dried oysters. The aroma compounds of VIP > 1 were regarded markers that can be used to distinguish among the samples. Additionally, four non-volatile flavors and eight aromas were screened as markers to differentiate among five samples: glutamic acid, glycine, betaine, IMP, pentanal, ethyl heptanoate, (E, Z)-2,4-nonadienal, 1-octen-3-one, 2-hexenal, 2-octenal, hexanal and decanal.

## 4. Conclusions

This study examined the effect of different drying methods on the flavor quality of oysters. The drying method was found to significantly affect the flavor characteristics of oysters. HAD strengthened the umami attributes of dried oysters, and VFD was superior in retaining umami nucleotides (AMP, IMP, GMP), whereas SD had the most prominent bitter and sweet taste. Compared with raw oysters, hot-digestion dry processing (HAD, VD, and NSD) enhanced the richness of dried oyster odor, imparting dried oysters with fruity, fatty, and roasted aromas. Moreover, glutamic acid, glycine, betaine, IMP, pentanal, ethyl heptanoate, (E, Z)-2,4-nonadienal, 1-octen-3-one, 2-hexenal, 2-octenal, hexanal, and decanal were defined as markers to distinguish among the drying methods. These results can serve as a basis for the standardization of dried-oyster production. Nevertheless, it is unclear how the characteristic flavor substances of dried oysters are produced. This involves a complex system of biochemical reactions, and elucidating the pathways of protein degradation, lipid thermal oxidation, and Maillard reactions during oyster drying in future studies will be important for improving the quality of dried aquatic products.

## Data Availability

The datasets generated for this study are available on request to the corresponding author.

## Supplementary Materials

The following supporting information can be downloaded at: https://www.mdpi.com/article/10.3390/foods12112136/s1, Table S1: Analysis of FAAs and flavor nucleotides in dried oysters under different drying conditions (mg/100 g, dry weight); Table S2: Taste attributes (+pleasant, −unpleasant) and TAVs in dried oysters under different drying conditions; Table S3: dentification and quantification of volatile compounds in dried oysters under different drying conditions (μg/100 g, dry weight); Table S4: Odor thresholds and aroma-active compounds in dried oysters under different drying conditions [41]; Table S5: Peak values of standards, regression equations, standard curve coefficients.
