# Supplementary material for: Effect of Drying Process on the Formation of the Characteristic Flavor of Oyster (Crassostrea hongkongensis)
Source: Foods. 2023 May 25;12(11):2136. doi: 10.3390/foods12112136 (PMC10253031; doi:10.3390/foods12112136)
Supplement: Supplementary file 1 [file foods-12-02136-s001.zip › foods-2363259-supplementary.pdf]

Supplementary Material

Contents:

Table S1. Analysis of FAAs and flavor nucleotides in dried oysters under different drying conditions(mg/100g, dry weight)

Notes: Different types of letters in the same row indicate significant differences (P<0.05); CK: blanched oysters; VFD: vacuum freeze drying; VD: vacuum drying; NSD: natural sun-drying; HAD: hot-air drying; ATP: adenosine triphosphate; ADP: adenosine 5′-pyrophosphate; IMP: inosine 5′-monophosphate; HXR: Inosine; HX: Inosine; AMP: adenosine 5′-monophosphate; GMP: guanosine 5′-monophosphate.

Table S2. Taste attributes (+pleasant, −unpleasant) and TAVs in dried oysters under different drying conditions

Notes: "—": not detected; different types of letters in the same row indicate significant differences (P<0.05); Threshold: from the literature [8, 15, 19]; CK: blanched oysters; VFD: vacuum freeze drying; VD: vacuum drying; NSD: natural sun-drying; HAD: hot-air drying; ATP: adenosine triphosphate; ADP: adenosine 5′-pyrophosphate; IMP: inosine 5′-monophosphate; HXR: inosine; HX: inosine; AMP: adenosine 5′-monophosphate; GMP: guanosine 5′-monophosphate.

Table S3. Identification and quantification (ug/100g) of volatile compounds in dried oysters under different drying conditions

Notes: RI: retention indices determined on a Pure-WAX; LD: literature data [18, 20, 21] and compared with published data previously reported in the published literature and listed in several credible online databases (http://www.flavornet.org; https://webbook.nist.gov); ID: identification method; MS: mass spectrum; RI: retention index; "—": not detected; different types of letters in the same row indicate significant differences (P<0.05); CK: blanched oysters; VFD: vacuum freeze drying; VD: vacuum drying; NSD: natural sun-drying; HAD: hot-air drying.

Table S4. Odor thresholds and aroma-active compounds in dried oysters under different drying conditions

Notes: Odor thresholds were referenced from a book named Odor thresholds compilations of odor threshold values in air, water and other media (second enlarged and revised edition) [41]; "—": not

detected; CK: blanched oysters; VFD: vacuum freeze drying; VD: vacuum drying; NSD: natural sun-drying; HAD: hot-air drying.

Table S5. Peak values of standards, regression equations, standard curve coefficients

Notes: Standard curves for nucleotides, organic acids, betaine, Y for concentration and X for peak area; Standard curve for amino acids, Y represents the peak area and X represents the concentration; ATP: adenosine triphosphate; ADP: adenosine 5′-pyrophosphate; IMP: inosine 5′-monophosphate; HXR: inosine; HX: inosine; AMP: adenosine 5′-monophosphate; GMP: guanosine 5′-monophosphate.

Table S1

Analysis of FAAs and flavor nucleotides in dried oysters under different drying conditions (mg/100g，dry weight)

Compounds	CK	VFD	VD	HAD	NSD
Aspartic acid	177.6±0.52^{b}	212.5±2.50^{a}	53.91±0.58^{e}	64.62±0.58^{d}	151.2±0.74^{c}
Threonine	126.04±1.04^{b}	47.50±1.51^{e}	63.28±0.78^{d}	89.2±0.19^{c}	138.92±0.44^{a}
Serine	94.27±0.52^{b}	29.50±0.51^{e}	47.66±0.87^{d}	53.48±0.09^{c}	128.9±0.58^{a}
Glutamic acid	333.85±0.55^{b}	191.5±1.20^{c}	60.94±1.56^{e}	677.67±1.57^{a}	72.12±0.64^{d}
Proline	42.19±0.72^{e}	60.25±0.25^{d}	76.31±0.22^{c}	89.29±3.57^{b}	153.36±2.39^{a}
Glycine	101.09±0.05^{e}	550.33±3.06^{a}	155.95±0.61^{d}	166.96±0.89^{c}	182.55±0.58^{b}
Alanine	171.88±5.21^{c}	120.25±0.25^{d}	445.31±7.81^{b}	466.33±2.62^{a}	447.81±2.33^{b}
Cystine	4.43±0.26^{d}	17.5±1.50^{a}	10.78±0.47^{b}	3.59±0.07^{e}	6.16±0.16^{c}
Valine	104.69±0.82^{b}	29.5±0.50^{e}	70.98±0.81^{c}	54.46±0.89^{d}	204.98±0.52^{a}
Methionine	68.23±0.52^{a}	5.75±0.25^{c}	5.47±0.16^{c}	4.82±0.18^{d}	7.34±0.38^{b}
Isoleucine	89.06±0.44^{b}	15.5±1.50^{e}	41.41±0.28^{c}	36.61±0.89^{d}	150.68±1.76^{a}
Leucine	167.19±1.12^{b}	37.5±2.50^{d}	63.28±0.78^{c}	61.61±2.68^{c}	247.5±1.07^{a}
Tyrosine	115.1±0.62^{b}	17.75±1.25^{e}	25.78±0.30^{d}	356.81±0.58^{a}	27.86±0.12^{c}
Phenylalanine	125.52±0.98^{a}	16.75±1.25^{e}	20.39±0.08^{d}	21.46±0.06^{c}	104.58±4.45^{b}
Lysine	146.35±0.52^{d}	152.5±2.50^{c}	187.19±0.6^{b}	143.75±0.89^{e}	236.25±0.54^{a}
Histidine	28.56±0.59^{b}	37.00±1.50^{a}	4.77±0.23^{e}	8.84±0.45^{d}	26.38±0.58^{c}
Arginine	51.41±0.55^{d}	280.25±0.25^{a}	66.95±0.67^{c}	83.04±0.89^{b}	35.36±1.07^{e}
Total amino acids	1947.46±10.32^{c}	1821.83±6.21^{d}	1400.35±5.65^{e}	2382.54±1.19^{a}	2321.94±6.02^{b}
ATP	3.73±0.18^{c}	3.25±0.21^{c}	4.66±0.25a^{b}	4.27±0.20b^{c}	5.17±0.09^{a}
ADP	14.99±0.50^{b}	58.71±2.21^{a}	13.72±0.99^{bc}	9.07±1.10^{c}	14.17±1.50^{bc}
IMP	10.94±0.78^{e}	113.36±2.89^{a}	43.51±1.44^{c}	63.96±1.66^{b}	20.08±1.01^{d}
AMP	11.5±0.47^{d}	107.56±1.79^{b}	79.39±3.59^{c}	190.67±3.37^{a}	85.17±5.67^{c}
HXR	83.8±3.23^{a}	33.65±0.29^{d}	47.91±2.10^{c}	47.21±1.76^{c}	74.04±3.23^{b}

HX	9.31±0.55[c]	62.96±2.50[a]	7.01±0.31[d]	4.24±0.89[e]	17.4±1.88[b]
GMP	37.77±1.58[a]	21.32±0.94[b]	10.66±0.46[e]	11.30±0.41[d]	20.18±1.01[c]
Total nucleotides	172.04±3.32[e]	400.80±15.79[a]	206.86±8.27[d]	330.71±6.57[b]	236.21±5.56[c]

Notes: Different types of letters in the same row indicate significant differences (P<0.05); CK: blanched oysters; VFD: vacuum freeze drying; VD: vacuum drying; NSD: natural sun-drying; HAD: hot-air drying; ATP: adenosine triphosphate; ADP: adenosine 5′-pyrophosphate; IMP: inosine 5′-monophosphate; HXR: Inosine; HX: Inosine; AMP: adenosine 5′-monophosphate; GMP: guanosine 5′-monophosphate.

Table S2

Taste attributes (+pleasant, −unpleasant) and TAVs in dried oysters under different drying conditions

**Compounds**	**Attribute**	**Threshold (mg/100g)**	**CK**	**VFD**	**VD**	**HAD**	**SD**
Aspartic acid	Umami (+)	100	1.78	2.13	< 1	< 1	1.51
Threonine	Sweet (+)	260	< 1	< 1	< 1	< 1	< 1
Serine	Sweet (+)	150	< 1	< 1	< 1	< 1	< 1
Glutamic acid	Umami (+)	30	11.13	6.38	2.03	22.59	2.40
Proline	Sweet/Bitter (+)	300	< 1	< 1	< 1	< 1	< 1
Glycine	Sweet (+)	130	< 1	4.23	1.20	1.28	1.40
Alanine	Sweet (+)	60	2.86	2.00	7.42	7.77	7.46
Cystine	Bitter/sweet (-)	—	—	—	—	—	—
Valine	Sweet/bitter (-)	40	2.62	< 1	1.77	1.36	5.12
Methionine	Bitter/sweet (-)	30	< 1	< 1	< 1	< 1	< 1
Isoleucine	Bitter (−)	90	< 1	< 1	< 1	< 1	1.67
Leucine	Bitter (−)	190	< 1	< 1	< 1	< 1	1.30
Tyrosine	Bitter (−)	—	—	—	—	—	—
Phenylalanine	Bitter (−)	90	1.39	< 1	< 1	< 1	1.16
Lysine	Sweet/bitter (−)	—	—	—	—	—	—
Histidine	Sulfurous (−)	20	1.43	1.85	< 1	< 1	1.32

Arginine	Bitter/Sweet (+)	50	< 1	5.61	1.34	1.66	0.71
ATP	—	—	—	—	—	—	—
ADP	—	—	—	—	—	—	—
IMP	Umami	25	< 1	4.53	1.61	2.53	< 1
AMP	Umami	50	< 1	2.22	1.59	3.81	1.70
HXR	—		—	—	—	—	—
HX	—		—	—	—	—	—
GMP	Umami	12.5	3.02	1.70	< 1	< 1	1.61

Notes: "—": not detected; different types of letters in the same row indicate significant differences ($P<0.05$); Threshold: from the literature [8, 15, 19]; CK: blanched oysters; VFD: vacuum freeze drying; VD: vacuum drying; NSD: natural sun-drying; HAD: hot-air drying; ATP: adenosine triphosphate; ADP: adenosine 5′-pyrophosphate; IMP: inosine 5′-monophosphate; HXR: inosine; HX: inosine; AMP: adenosine 5′-monophosphate; GMP: guanosine 5′-monophosphate.

Table S3

Identification and quantification of volatile compounds in dried oysters under different drying conditions (ug/100g， dry weight)

Compounds	RI	LD	ID	CK	VD	VFD	HAD	SD
hexanal	1,085	1084	MS, RI	0.75±0.19^{e}	25.51±1.98^{c}	66.65±1.92^{a}	39.79±1.05^{b}	12.38±5.38^{d}
octanal	1,283	1268	MS, RI	1.42±0.14^{c}	28.62±2.37^{a}	26.39±3.86^{a}	29.95±0.02^{a}	10.05±3.17^{b}
heptanal	984	993	MS, RI	1.02±0.55^{d}	22.07±1.49^{b}	25.87±1.10^{a}	24.83±0.68^{a}	8.18±3.63^{c}
(E, E)-2,4-heptadienal	1,372	1360	MS, RI	9.87±2.49^{e}	45.88±0.83^{b}	28.85±6.34^{d}	91.15±3.99^{a}	42.07±1.66^{c}
(e)-2-decenal	1,571	1503	MS, RI	1.49±0.09^{d}	10.14±0.75^{c}	16.16±1.21^{b}	27.17±2.88^{a}	10.8±0.38^{c}
(E, E)-2,4-decadienal	1,780	1774	MS, RI	0.86±0.09^{c}	5.69±2.34^{b}	—	15.01±3.65^{a}	13.06±1.01^{a}
4-pentenal	1,220	1134	MS, RI	0.24±0.04^{c}	2.05±0.14^{b}	1.93±0.46^{b}	4.51±1.62^{a}	3.12±0.16^{a}
nonanal	1,386	1370	MS, RI	1.96±0.21^{d}	16.96±1.45^{b}	20.27±2.18^{a}	16.65±0.44^{b}	5.33±1.16^{c}
tridecanal	1,305	1313	MS, RI	0.3±0.02^{c}	2.23±1.19^{b}	—	9.1±2.85^{a}	—
cinnamaldehyde	2,287	2001	MS, RI	0.11±0.01^{b}	0.60±0.18^{a}	—	0.87±0.07^{a}	—
pentanal	998	985	MS, RI	—	3.66±1.48^{c}	10.92±1.30^{a}	7.65±0.41^{b}	5.14±0.26^{c}
2-hexenal	1,120	1103	MS, RI	—	6.69±1.44^{b}		11.21±1.41^{a}	3.36±0.85^{c}
2-octenal	1,412	1408	MS, RI	4.44±0.38^{d}	20.97±1.34^{c}	33.17±0.82^{a}	27.42±0.66^{b}	—
(E, E)-2,4-octadienal	1,740	1605	MS	—	—	—	4.44±1.48^{a}	3.61±0.33^{a}
(E, Z)-2,4-nonadienal	1,666	1709	MS, RI	5.37±0.33^{c}	15.74±2.10^{b}	—	35.38±4.20^{a}	15.35±0.35^{b}
(E)-2-dodecenal	1409	1386	MS, RI	—	—	—	6.74±0.79^{a}	6.2±0.83^{a}
benzaldehyde	1,493	1477	MS, RI	2.46±0.02^{e}	30.29±3.38^{c}	24.49±0.87^{d}	41.21±0.53^{a}	37.49±1.40^{b}
decanal	1,512	1507	MS, RI	0.68±0.08^{c}	8.84±0.37^{a}	4.5±1.22^{b}	—	—
hexadecanal	1,960	1960	MS, RI	—	2.32±1.14^{a}	—	—	3.45±0.34^{a}
4-ethyl-benzaldehyde	2,038	—	MS	—	7.40±1.65^{b}	—	5.41±0.09^{b}	15.54±0.78^{a}
2-heptanone	1,153	1162	MS, RI	—	5.65±0.30^{a}	—	5.31±0.36^{a}	4.26±0.11^{b}
1-octen-3-one	1,335	1331	MS, RI	—	6.21±0.44^{a}	—	1.35±0.05^{c}	4.55±0.21^{b}
3,5-octadiene-2-one	1,501	1537	MS, RI	—	—	6.17±0.76^{b}	9.26±0.98^{a}	—

2,3-pentanedione	1,365	1508	MS	—	2.12±1.58^{a}	—	0.84±0.27^{b}	2.51±1.76^{a}
3,5-octadien-2-one	1,699	1689	MS, RI	1.92±0.04^{d}	12.37±0.46^{a}	7.76±2.73^{c}	11.25±0.13^{b}	13.83±1.37^{a}
2-tridecanone	1,787	1755	MS, RI	—	7.43±0.90^{b}	—	9.66±1.30^{a}	10.26±0.43^{a}
3-nonen-2-one	1,506	1494	MS, RI	—	—	5.61±0.48^{a}	1.75±0.56^{b}	—
2-methyl-cyclopentanone	2,183	—	MS	—	—	9.55±0.95^{a}	—	2.03±0.24^{b}
2-butanone	1,002	947	MS, RI	—	6.78±0.18^{b}	—	7.65±0.19^{a}	4.65±0.23^{c}
2-octanone	1,249	1244	MS, RI	2.28±0.3	—	—	—	—
2,3-octanedione	1,318	1338	MS, RI	0.88±0.64	—	—	—	—
2-pentadecenone	1,954	1999	MS, RI	4.54±0.14^{a}	3.78±2.83^{a}	—	—	—
1-pentene-3-ol	1,203	1148	MS, RI	1.35±0.27^{c}	—	3.39±0.33^{b}	4.75±0.06^{a}	—
pentadecanol	1,623	1644	MS, RI	0.45±0.00^{c}	6.45±1.09^{b}	12.02±0.88^{a}	8.31±1.27^{b}	—
1-octanol	1,353	1428	MS, RI	0.58±0.07^{b}	—	—	3.7±0.01^{a}	—
6-methyl-1-heptanol	1,360	1524	MS, RI	—	—	—	0.69±0.24^{b}	3.46±0.73^{a}
1-octene-3-ol	1,430	1464	MS, RI	—	17.21±5.01^{a}	19.78±0.69^{a}	5.34±0.76^{c}	—
hepten-3-ol	1,190	1322	MS,RI	—	—	—	2.58±0.85^{b}	7.22±0.25^{a}
heptanol	1,268	1232	MS, RI	—	7.83±1.90	—	—	—
methyl pentadecanoate	1,970	2108	MS, RI	—	—	—	2.77±0.48^{a}	3.12±0.13^{a}
methyl-2-methylbutyrate	1,410	1387	MS, RI	1.03±0.09^{b}	—	—	1.64±0.60^{b}	9.01±0.57^{a}
ethyl heptanoate	2,160	2202	MS, RI	0.96±0.01^{c}	8.83±2.56^{a}	1.37±0.06^{b}	—	—
ethyl propanoate	1,886	1991	MS, RI	—	—	—	3.25±0.16^{a}	2.17±0.10^{c}
ethyl hexanoate	1,620	1634	MS, RI	—	—	—	2.45±0.32	—
n-caproic acid vinyl ester	1,737	—	MS	—	8.11±0.98^{a}	—	3.25±0.45^{b}	—
octanoic acid	2,034	2024	MS, RI	—	3.43±0.27^{a}	4.45±1.24^{a}	4.26±1.09^{a}	—
nonanoic acid	2,360	2251	MS, RI	—	1.4±0.17^{b}	6.33±1.49^{a}	1.29±0.54^{b}	7.31±0.68^{a}
3-methylbutanoic acid	1,665	1639	MS, RI	—	—	—	—	9.66±0.87
hexanoic acid	1,603	1642	MS, RI	—	9.46±0.74^{a}	—	—	3.32±1.52^{b}

heptanoic acid	1,902	1960	MS,RI	—	2.59±0.12^{b}	9.36±0.52^{a}	—	—
2-pentylfuran	1,231	1230	MS, RI	0.75±0.02^{c}	9.88±1.08^{b}	—	7.11±0.28^{b}	10.31±1.69^{a}
2-5-dimethylfuran	911	936	MS, RI	—	11.03±0.14^{b}	—	15.32±1.03^{a}	8.45±0.13^{c}
2-ethylfuran	976	955	MS, RI	—	5.11±1.27^{c}	5.01±0.37^{c}	13.5±0.59^{b}	27.13±5.81^{a}
trans-2-(2- pentenyl) furan	1,248	1282	MS, RI	—	17.72±1.81^{b}	24.39±1.99^{a}	14.54±0.43^{c}	—
2-acetylfuran	1,495	1498	MS. RI	—	—	—	3.96±0.14b	5.99±1.30a
2-acetylpyrrole	1,909	1919	MS, RI	—	0.33±0.05^{c}	3.92±0.31^{a}	1.28±0.08^{b}	—
octadecane	1,482	1425	MS, RI	1.21±0.01^{b}	—	—	—	11.19±0.41^{a}
2,6,10-trimethyldodecane	1,721	1784	MS, RI	1.95±0.05^{c}	—	—	7.08±1.77^{b}	16.27±1.82^{a}
(Z)-5-tetradecen-3-yne	1,500	1359	MS, RI	2.55±0.50^{b}	10.24±1.65^{a}	—	—	—
3-ethyl-2-methyl-1,3-hexadiene	1,050	1041	MS, RI	1.55±0.07^{b}	3.13±0.53^{a}	—	—	—
Total volatile substances				52.97±6.85^{d}	422.75±21.68^{b}	378.31±34.08^{bc}	552.63±41.81^{a}	361.83±22.05^{c}

Notes: RI: retention indices determined on a Pure-WAX; LD: literature data [18, 20, 21] and compared with published data previously reported in the published literature and listed in several credible online databases (http://www.flavornet.org; https://webbook.nist.gov); ID: identification method; MS: mass spectrum; RI: retention index; "—": not detected; different types of letters in the same row indicate significant differences (*P*<0.05); CK: blanched oysters; VFD: vacuum freeze drying; VD: vacuum drying; NSD: natural sun-drying; HAD: hot-air drying.

Table S4

Odor thresholds and aroma-active compounds in dried oysters under different drying conditions

compounds	threshold(ug/100g)	odorant description	OAV				
			CK	VD	VFD	HAD	SD
hexanal	0.50	green, fatty	1.50	51.02	133.30	79.58	24.76
octanal	0.17	mint, fatty	8.35	168.35	155.24	176.18	59.12
heptanal	1.30	green, fruity	< 1	16.98	19.90	19.10	6.29
(E, E)-2,4-heptadienal	1.54	nutty, fatty	6.41	29.79	18.73	59.19	27.32
(E)-2-decenal	0.03	oils	49.67	338.00	538.67	905.67	360.00
(E, E)-2,4-decadienal	0.05	fatty	17.20	113.80	—	300.20	261.20
nonanal	0.10	fatty, fishy	19.60	169.60	202.70	166.50	53.30
cinnamaldehyde	6.90	cinnamon	< 1	< 1	—	< 1	—
pentanal	0.14	green, fruity	—	26.14	78.00	54.64	36.71
2-hexenal	1.70	green, fruity	—	3.94		6.59	1.98
2-octenal	0.30	nutty, fatty	14.80	69.90	110.57	91.40	—
(E, Z)-2,4-nonadienal	19.00	fruity, fatty	< 1	< 1	—	1.86	< 1
benzaldehyde	35.00	almond, smoke	< 1	< 1	< 1	1.18	1.07
decanal	5.00	fatty, orange	< 1	1.77	< 1	—	—
1-octen-3-one	0.40	mushroom	—	15.53	—	3.38	11.38
2,3-pentanedione	0.10	cream	—	21.20	—	8.40	25.10
1-pentene-3-ol	40.00	—	< 1	—	< 1	< 1	—
1-octanol	0.54	waxy, orange	< 1	—	—	< 1	—
1-octene-3-ol	0.15	mushroom, earthy	—	114.73	131.87	35.60	—
ethyl heptanoate	0.20	fruity	4.80	44.15	6.85	—	—

ethyl hexanoate	0.50	almond, apple	—	—	—	4.90	—
nonanoic acid	300.00	fatty, cheese	—	< 1	< 1	< 1	< 1
hexanoic acid	300.00	fruity, fatty	—	< 1	—	—	< 1
heptanoic acid	30.00	fruity, fatty	—	< 1	< 1	—	—
2-pentylfuran	0.58	caramel, coffee	1.29	17.03	—	12.26	17.78
2-acetylfuran	100.00	caramel, coffee	—	< 1	—	< 1	< 1
2-ethylfuran	800.00	burnt, coffee, chocolate	—	< 1	< 1	< 1	< 1

Notes: Odor thresholds were referenced from a book named Odor thresholds compilations of odor threshold values in air, water and other media (second enlarged and revised edition) [41]; "—": not detected; CK: blanched oysters; VFD: vacuum freeze drying; VD: vacuum drying; NSD: natural sun-drying; HAD: hot-air drying.

Table S5

Peak values of standards, regression equations, standard curve coefficients

Standards	Peak areas for different standard concentrations					Regression equation	R^2
	20μg/g	40μg/g	60μg/g	80μg/g	100μg/g		
ATP	1381.20	2781.80	4141.10	5437.00	6981.20	$y= 0.0145x + 0.089$	$R^2=0.9993$
ADP	1082.80	2168.20	3195.00	4238.50	5418.70	$y = 0.0187x + 0.0133$	$R^2=0.9994$
IMP	589.80	1163.20	1727.30	2276.60	2936.80	$y = 0.0344x + 0.0867$	$R^2=0.9994$
Hx	1277.30	2547.30	3851.60	5107.80	6550.90	$y = 0.0154x + 0.4852$	$R^2=0.9994$
AMP	915.40	1813.30	2742.30	3645.10	4674.70	$y = 0.0216x + 0.4909$	$R^2=0.9994$
HxR	1170.40	2320.70	3513.80	4678.00	6000.00	$y = 0.0168x + 0.5547$	$R^2=0.9994$
GMP	453.37	935.30	1417.23	1855.15	2381.08	$y = 0.0415x + 1.8151$	$R^2=0.9992$
	100.00μg/g	200.00μg/g	400.00μg/g	600.00μg/g	1000.00μg/g		
Malic acid	105.10	202.80	397.50	586.80	990.80	$y = 1.0182x - 4.912$	$R^2=0.9998$
Citric acid	623.50	1220.60	2277.80	3600.60	6022.40	$y = 0.1662x + 3.0734$	$R^2=0.9991$
Lactic acid	100.00	200.00	400.00	600.00	1000.00	$y = 1.0397x + 5.7809$	$R^2=0.9990$
Succinic acid	87.10	170.80	318.90	504.70	845.60	$y = 1.1831x + 4.0194$	$R^2=0.9991$
Betaine	82.02	163.15	325.41	480.67	812.19	$y=12.3226x-11.0231$	$R^2=0.9999$
	0.10μg/g	0.20μg/g	0.40μg/g	0.60μg/g	1.00μg/g		
Aspartic acid	25.08	63.34	136.87	216.39	369.44	$y = 382.62x - 13.183$	$R^2=0.9999$
Glutamic acid	24.89	62.93	139.01	211.09	367.24	$y = 380.39x - 13.146$	$R^2=0.9998$
Serine	29.01	70.17	152.50	227.83	401.49	$y = 411.64x - 12.154$	$R^2=0.9995$
Glycine	39.22	80.13	160.94	245.75	407.37	$y = 409.05x - 1.6833$	$R^2=0.9998$
Histidine	29.09	71.74	153.05	245.35	412.95	$y = 426.51x - 13.558$	$R^2=0.9996$
Arginine	28.27	65.52	155.01	234.49	399.47	$y = 412.44x - 12.971$	$R^2=0.9997$
Threonine	15.08	56.79	130.23	213.66	372.53	$y = 397.17x - 24.642$	$R^2=0.9991$

Alanine	26.69	68.48	153.06	230.65	402.82	y = 417.92x - 15.104	R^2=0.9998
Proline	37.62	80.94	164.57	254.21	427.48	y = 433.18x - 5.700	R^2=0.9999
Tyrosine	33.13	78.25	163.50	258.74	439.22	y = 451.21x - 11.988	R^2=0.9998
Valine	33.22	78.69	164.63	260.57	442.45	y = 454.70x - 12.250	R^2=0.9998
Methionine	31.11	74.99	162.74	245.49	425.99	y = 438.76x - 12.767	R^2=0.9998
Isoleucine	32.39	76.22	167.89	248.56	426.89	y = 438.34x - 11.446	R^2=0.9998
Leucine	36.69	83.76	165.90	257.04	433.32	y = 440.70x - 7.3833	R^2=0.9998
Phenylalanine	40.64	79.80	172.12	262.43	443.07	y = 451.59x - 8.5208	R^2=0.9998
Lysine	50.48	133.36	269.12	414.88	706.39	y = 728.79x - 22.396	R^2=0.9998
Cystine	19.09	50.47	128.24	192.02	328.56	y = 343.86x - 15.700	R^2=0.9996

Notes: Standard curves for nucleotides, organic acids, betaine, Y for concentration and X for peak area; Standard curve for amino acids, Y represents the peak area and X represents the concentration; ATP: adenosine triphosphate; ADP: adenosine 5′-pyrophosphate; IMP: inosine 5′-monophosphate; HXR: inosine; HX: inosine; AMP: adenosine 5′-monophosphate; GMP: guanosine 5′-monophosphate.
